# Circulating Immune Complexes among Diabetic Children

**DOI:** 10.1080/10446670410001670517

**Published:** 2004-03

**Authors:** George Nicoloff, Alexander Blazhev, Chaika Petrova, Petkana Christova

**Affiliations:** 1 Department of Biology and Pathological Physiology University School of Medicine Pleven Bulgaria; 2 Department of Pediatric University School of Medicine Pleven Bulgaria; 3 Department of Social Medicine University School of Medicine Pleven Bulgaria; 4 Division of Biology Department of Biology and Pathological Physiology University School of Medicine 1, St. Kliment Ohridski Street Pleven 5800 Bulgaria

## Abstract

Insulin dependent diabetes mellitus (IDDM) is an autoimmune disease associated with the presence of different types of autoantibodies. The presence of these antibodies and the corresponding antigens in the circulation leads to the formation of circulating immune complexes (CIC). CIC are known to persist in the blood for long periods of time. Such CIC following deposition in the small blood vessels have the potential to lead to microangiopathy with debilitating clinical consequences. The aim of our pilot study was to investigate whether a correlation exists between CIC and the development of microvascular complications in diabetic children. Isolation of a new glycoprotein complement inhibition factor (CIF) from the parasitic plant *Cuscuta europea* seed, which appears to bind specifically to complement component C3 has provided an unique tool for the measurement of immune complexes by means of ELISA-type techniques (CIF-ELISA). We studied the levels of CIC (IgG, IgM and IgA) in 58 diabetic children (mean age 12.28±4.04 years, diabetes duration 5.3±3.7 years), 29 of them had vascular complications (group 1) and the other 29 were without vascular complications (group 2). As controls, we studied sera samples from 21 healthy children (mean age 13.54±4.03 years). Sera from the diabetic patients showed statistically significant higher levels of CIC IgG ( *p*=0.03) than sera from the control group. In sera from group 1 values of CIC IgG showed statistically significant higher levels than controls (0.720±0.31 vs. 0.46±0.045; p*=*0.011) Sera from 59% of the patients were positive for CIC IgG, 36% for CIC IgM and 9% for CIC IgA. Among 26 patients with microalbuminuria, sera from 17/26 (65%) were positive for CIC IgG, 8/26 (31%) for CIC IgM and 2/26 (8%) for CIC IgA. CIC IgG correlated with HbA1c (*r*=0.51; *p*=0.005) and microalbuminuria (*r*=0.42, *p*=0.033). CIC IgA correlated with age (*r*=0.44, *p*=0.03). CIC IgM correlated with the duration of diabetes (*r*=0.63, *p*=0.02). These findings suggest that elevated levels of CIC IgG are associated with the development of early diabetic nephropathy.


*Clinical & Developmental Immunology*, March 2004, VOL. 11 (1), pp. 61–66

Taylor&Francis healthsciences

### Circulating Immune Complexes among Diabetic Children

GEORGE NICOLOFF[a,*], ALEXANDER BLAZHEV[a], CHAIKA PETROVA[b] and PETKANA CHRISTOVA[c]

[a]*Department of Biology and Pathological Physiology, University School of Medicine, Pleven, Bulgaria;* [b]*Department of Pediatric, University School of Medicine, Pleven, Bulgaria;* [c]*Department of Social Medicine, University School of Medicine, Pleven, Bulgaria*

Insulin dependent diabetes mellitus (IDDM) is an autoimmune disease associated with the presence of different types of autoantibodies. The presence of these antibodies and the corresponding antigens in the circulation leads to the formation of circulating immune complexes (CIC). CIC are known to persist in the blood for long periods of time. Such CIC following deposition in the small blood vessels have the potential to lead to microangiopathy with debilitating clinical consequences. The aim of our pilot study was to investigate whether a correlation exists between CIC and the development of microvascular complications in diabetic children. Isolation of a new glycoprotein complement inhibition factor (CIF) from the parasitic plant *Cuscuta europea* seed, which appears to bind specifically to complement component C3 has provided an unique tool for the measurement of immune complexes by means of ELISA-type techniques (CIF-ELISA). We studied the levels of CIC (IgG, IgM and IgA) in 58 diabetic children (mean age $12.28 \pm 4.04$ years, diabetes duration $5.3 \pm 3.7$ years), 29 of them had vascular complications (group 1) and the other 29 were without vascular complications (group 2). As controls, we studied sera samples from 21 healthy children (mean age $13.54 \pm 4.03$ years). Sera from the diabetic patients showed statistically significant higher levels of CIC IgG ($p = 0.03$) than sera from the control group. In sera from group 1 values of CIC IgG showed statistically significant higher levels than controls ($0.720 \pm 0.31$ vs. $0.46 \pm 0.045$; $p = 0.011$) Sera from 59% of the patients were positive for CIC IgG, 36% for CIC IgM and 9% for CIC IgA. Among 26 patients with microalbuminuria, sera from 17/26 (65%) were positive for CIC IgG, 8/26 (31%) for CIC IgM and 2/26 (8%) for CIC IgA. CIC IgG correlated with HbA1c ($r = 0.51$; $p = 0.005$) and microalbuminuria ($r = 0.42$, $p = 0.033$). CIC IgA correlated with age ($r = 0.44$, $p = 0.03$). CIC IgM correlated with the duration of diabetes ($r = 0.63$, $p = 0.02$). These findings suggest that elevated levels of CIC IgG are associated with the development of early diabetic nephropathy.

*Keywords*: Diabetes mellitus; CIF-ELISA; Circulating immune complexes; Microalbuminaria

### INTRODUCTION

Immune complexes are a heterogeneous high molecular weight aggregates composed of antigens and immunoglobulins elicited in response to antigens, and in most cases, include complement component C3 (Stanilova and Slavov, 2001). When immune complexes are produced faster than they can be cleared from the circulation, immune complex disease may occur. Examples of such disorders include drug sensitivity, rheumatoid arthritis and systemic lupus erythematosus (SLE). The presence of detectable immune complexes in the blood is considered important for the diagnosis of autoimmune diseases in general.

Type 1 (insulin-dependent) diabetes mellitus results from the progressive autoimmune destruction of pancreatic beta cells (Bottazzo, 1993). On the other hand, an important factor for the development of diabetic vascular complications is the alteration in the structure of the vascular proteins. Peptides derived from such processes have been found in the circulation (Nicoloff *et al.*, 2000a, 2001) and they are reasoned to be a stimulus for increased production of autoantibodies. A variety of autoantibodies have been documented in diabetes and include insulin autoantibodies, GAD-autoantibodies, tyrosine phosphatase IA2-autoantibodies, islet cell-autoantibodies, (Verge *et al.*, 1998) elastin autoantibodies (Nicoloff *et al.*, 2000b) and collagen type IV autoantibodies (Nicoloff *et al.*, 2002). These autoantibodies bind to their cognate antigen and thus form circulating immune complexes (CIC). CIC could persist in the blood for a long time. Such CIC have pathogenic potential since following deposition on small blood vessels they give rise to microangiopathy.

Recently, the identification of a new glycoprotein termed the complement inhibition factor (CIF) isolated from the parasitic plant *Cuscuta europea* seed, which appears to bind specifically to complement component

*Corresponding author. Address: Division of Biology, Department of Biology and Pathological Physiology, University School of Medicine, 1, St. Kliment Ohridski Street, 5800 Pleven, Bulgaria. Tel.: +359-64-884271. Fax: +359-64-801-603. E-mail: nicoloff_bg@yahoo.com

ISSN 1740-2522 print/ISSN 1740-2530 online © 2004 Taylor & Francis Ltd
DOI: 10.1080/10446670410001670517

C3 (Zhelev *et al.*, 1994) has provided a novel method for the detection and quantitation of immune complexes detection using standard ELISA based techniques (CIF-ELISA) (Stanilova and Slavov, 2001). The aim of our pilot study is to investigate utilizing this newly defined CIF-ELISA the levels of CIC (IgG, IgM and IgA). To achieve this objective we studied a total of 58 diabetic children. The data obtained on the samples from these patients were compared with a control group of 21 otherwise healthy age matched children.

### MATERIALS AND METHODS

### Subjects

The baseline study population consisted of a total of 58 patients (25 boys and 33 girls) with Type 1 diabetes mellitus (mean age $12.28 \pm 4.04$ years) diagnosed according to the WHO definition and for purposes of control a group of 21 healthy children of similar age ($13.54 \pm 4.03$) and sex with no family history of diabetes, atherosclerosis, and/or nephropathy. The patients are all diabetic children residing in the vicinity of the Pleven University Hospital. All patients were treated with a standard dose of human insulin obtained from Novo Nordisk Industri, Copenhagen, Denmark. None of the subjects were taking anti-hypertensive medication prior to the appearance of persistent microalbuminuria. None of the patients had a diagnosis of renal disease unrelated to diabetes.

The mean duration of diabetes was $5.3 \pm 3$ years. All subjects were being administered 2–4 subcutaneous doses of insulin per day. Twenty-nine of the 58 diabetics developed vascular complications. Of these 29 patients, 19 showed evidence of microalbuminuria, 3 developed high blood pressure, 2 developed both retinopathy and microalbuminuria, 4 developed both microalbuminuria and high blood pressure and 1 microalbuminuria, high blood pressure and retinopathy (see Table I).

Microalbuminuria was defined as a persistent urinary albumin excretion rate (AER) in the range of 20–200 μg/min in urine. Levels of hemoglobin A1c (HbA1c), total serum cholesterol, triglycerides and AER were measured as described earlier (Nicoloff *et al.*, 2000a). Levels of HDL were quantitated using the quantitative enzyme method for direct detection (test-kits HDL-C plus, Roche).

Ethical approval was obtained from the local research ethics committee and the parents of all subjects gave a written informed consent prior to enrolment in the study.

### Procedure

Isolation of CIF was performed according to the protocol developed by Stanilova and Slavov (2001). CIF was isolated from *C. europea* seeds (Zhelev *et al.*, 1994).

CIF-ELISA-flat bottom polystyrene microtiter plates were coated with 20 μg/ml CIF in 0.2 M carbonate–bicarbonate buffer pH 9.6 and incubated overnight at 4°C. After washing with washing buffer which consisted of 50 mM Tris-HCl pH 7.2 containing 0.05% Tween 20 ($2 \times 5$ min), the plates were blocked for 20 min with the same buffer. One hundred microlitres of 1:50 dilution of each serum to be tested in sample buffer (0.1 M PBS, containing 1% BSA 0.2% Tween 20, 1 mM $CaCl_2$ and $MgCl_2$) was added to duplicate wells of the microtiter plate and incubated for 60 min at 37°C. The microtiter wells were washed twice with washing buffer and then 100 μl of a 1:800 dilution of a peroxidase conjugated sheep anti-human IgG, IgM and IgA in washing buffer were added to appropriate wells. The microtiter plate was incubated for 60 min at 37°C. The reaction was stopped by the addition of 50 μl 8 N $H_2SO_4$. The O.D. 492 reading of the wells was then determined using a Microelisa Plate Reader 210 (Organon Teknika, Belgium).

### Statistical Analyses

All values are expressed as mean ± SD. Statistical analyses were performed using the computer programs Excel and Statgraphics plus for Windows. The Student's *t*-test and ANOVA were used to assess differences between study groups. For select data sets correlation analysis was performed and data considered significant with a $p$ value of less than 0.05.

TABLE I CIC in serum of children with Type 1 diabetes mellitus with or without vascular complications (VC)

| | | CIC | | Positive for CIC | | |
|---|---|---|---|---|---|---|
| Group | Sample size | Positive | Negative | IgG | IgM | IgA |
| Diabetics | 58 | 44 | 14 | 34/58 | 23/58 | 5/58 |
| Without VC | 29 | 21 | 8 | 14/29 | 14/29 | 3/29 |
| M | 19 | 16 | 3 | 14/19 | 8/19 | 1/19 |
| M + HBP | 4 | 2 | 2 | 2/4 | 0/4 | 0/4 |
| M + R | 2 | 1 | 1 | 0/2 | 0/2 | 1/2 |
| M + R + HBP | 1 | 1 | 0 | 1/1 | 0/1 | 0/1 |
| HBP | 3 | 3 | 0 | 3/3 | 1/3 | 0/3 |

The table shows the frequency of samples positive for CIC (IgG, IgM and IgA) in each group.
VC, vascular complications; M, microalbuminuria; R, retinopathy; HBP, high blood pressure.

TABLE II Clinical characteristics of diabetic patients with or without vascular complications

| CLINICAL DATA | Group 1 (with vascular complications) $n = 29$ | Group 2 (without vascular complications) $n = 29$ | $P$ |
|---|---|---|---|
| Age (years) | 14.03 ± 3.6 | 10.5 ± 3.9 | 0.0007 |
| Mean duration (years) | 6.9 ± 3.2 | 3.6 ± 3.43 | 0.0004 |
| Mean glycated hemoglobin(%) | 10.35 ± 1.6 | 10.4 ± 1.3 | NS |
| Systolic blood pressure (mmHg) | 110.3 ± 14.3 | 104.8 ± 11.2 | NS |
| Diastolic blood pressure (mmHg) | 75.5 ± 11.1 | 68.8 ± 7.9 | 0.01 |
| Triglycerides (mmol/l) | 1.37 ± 0.58 | 1.14 ± 0.57 | NS |
| Total cholesterol (mmol/l) | 4.71 ± 0.84 | 4.82 ± 0.76 | NS |
| Dose (U/kg/24 h) | 1.11 ± 0.29 | 0.85 ± 0.28 | 0.0011 |
| Microalbuminuria (μg/min) | 37.76 ± 16.9 | 10.14 ± 3.18 | 0.0001 |
| High density lipoprotein (mmol/l) | 1.33 ± 0.34 | 1.16 ± 0.19 | NS |
| CIC IgG ($E^{492}$) | 0.72 ± 0.31 | 0.62 ± 0.29 | NS |
| CIC IgM ($E^{492}$) | 0.14 ± 0.05 | 0.15 ± 0.04 | NS |
| CIC IgA ($E^{492}$) | 0.16 ± 0.04 | 0.16 ± 0.04 | NS |

### RESULTS

The clinical data of the tested patients are presented in Table II. The patients were divided into two groups based on the presence (group 1) or absence (group 2) of vascular complications. The mean ± 2 S.D. value was calculated on data obtained on sera samples from the control study group in efforts to define the normal range of CIC (IgG, IgM, IgA) and a cut-off value for classifying a sample as being positive. The results of this analysis are presented in Table III. As seen, diabetics showed statistically significant higher levels of CIC IgG than controls (0.672 ± 0.300 vs. 0.46 ± 0.045; $p = 0.03$). While the CIC IgG values obtained on samples from Group 1 were not significantly different from the group 2 (Table II), CIC IgG values obtained from groups 1 and 2 differed in terms of their significance to control values. Thus, while values of CIC IgG were statistically significant as compared with controls (0.720 ± 0.31 vs. 0.46 ± 0.045; $p = 0.011$), values obtained on samples from group 2 did not show statistically significant difference as compared with controls (0.62 ± 0.29 vs. 0.46 ± 0.045; $p = 0.28$). Thirty-four (59%) of the patients were positive for CIC IgG and 21 (36%) for CIC IgM. Five (9%) of the patients were positive for CIC IgA (Table I). From the 34 positive samples for CIC IgG 23 (68%) belonged to group1 and 14 (41%) to group 2. From the 23 positive samples for CIC IgM, 9 (39%) belonged to group 1 and 14 (61%) to group 2. Seventeen patients with microalbuminuria were positive for CIC IgG, 8 for CIC IgM and 2 for IgA. One of three diabetics with retinopathy had raised level of CIC IgG. Eight patients had high blood pressure, six of them were positive for CIC IgG and 1 for CIC IgM. CIC IgG of all patients was associated with diastolic blood pressure ($r = 0.29$, $p = 0.032$). Values for CIC IgG in the patients with vascular complications correlated with values of HbA1c ($r = 0.51$; $p = 0.005$) and microalbuminuria ($r = 0.42$, $p = 0.033$). While the values for CIC IgA correlated with age ($r = 0.44$, $p = 0.03$), the values of CIC IgM correlated with the duration of diabetes ($r = 0.63$, $p = 0.02$). The $p$ values for differences between groups 1 and 2 in terms of age ($p = 0.0007$), mean diabetes duration ($p = 0.0004$), diastolic blood pressure ($p = 0.01$), insulin dose ($p = 0.001$) and microalbuminuria ($p = 0.0001$) are shown in Table II.

TABLE III Study of the levels of circulating immune complexes (CIC IgG, CIC IgM, CIC IgA) in diabetics and control sera using CIF-ELISA

| | IgG | | IgM | | IgA | |
|---|---|---|---|---|---|---|
| | X | SD | X | SD | X | SD |
| Diabetics | 0.672 | 0.300 | 0.143 | 0.045 | 0.160 | 0.042 |
| Controls | 0.46 | 0.045 | 0.124 | 0.015 | 0.137 | 0.035 |
| X ± 2S.D. | 0.55 | 0.37 | 0.154 | 0.094 | 0.207 | 0.067 |

### DISCUSSION

In the present study, children with Type 1 diabetes mellitus were studied for levels of isotype specific immune complexes utilizing a newly developed ELISA assay. We chose children as a subject for the study because in adults CIC occur due to a variety of different pathological reasons. Using diabetic children it was easier to exclude the other factors (except diabetes) which lead to the formation of CIC. Individuals with juvenile onset Type 1 diabetes mellitus are at a high risk for the development of diabetic microangiopathy and vascular disease (Linderkamp *et al.*, 1999).

Similar studies on the quantitation of CIC levels have been conducted on a variety of autoimmune disease including systemic sclerosis (Stanilova and Slavov, 2003), SLE and rheumatoid arthritis (Ölmez *et al.*, 1991; Stanilova and Slavov, 2001). Type 1 diabetes mellitus is also an autoimmune disease in which different types of autoantibodies have been identified such as those against beta cells (insulin-autoantibodies, islet cell-autoantibodies, GAD autoantibodies (Verge *et al.*, 1998)) and antibodies against vascular wall proteins such as elastin (Nicoloff *et al.*, 2000b) and collagen (Nicoloff *et al.*, 2002). Our aim in the studies reported herein was to test the relationship between CIC and vascular complications in diabetic children.

Our laboratory has previously reported results of ELISA based specific titers of elastin-specific CIC (Baydanoff *et al.*, 1988) using sequential precipitation of CIC by increasing concentrations of polyethylene glycol (PEG). This method is not only time consuming but also labor intensive as compared to the CIF-ELISA described herein. As noted in the present study, the values for CIC in samples from adult diabetics with vascular complications were markedly higher than healthy controls.

This relationship between vascular complications and levels of CIC in the sera of diabetic patients has also been reported by others (Adreani *et al.*, 1982; Di Mario *et al.*, 1983; Baranao *et al.*, 1985; Baydanoff *et al.*, 1988; Turk *et al.*, 2001). Thus, while some studies reported a correlation between vascular complications and the detection of elastin-specific CIC (Baydanoff *et al.*, 1988), others have reported AGE-containing CIC (Turk *et al.*, 2001), insulin-CIC (Triolo *et al.*, 1984), and/or total CIC (Adreani *et al.*, 1982; Di Mario *et al.*, 1983; Baranao *et al.*, 1985). In the present study we investigated total CIC. Andreani *et al.* (1982) used a solid phase C1q (C1q SP) binding test and conglutinin radioimmune assay (RIA) for the detection of CIC. The results obtained using the C1qSP method showed both an increase in the prevalence and quantity of CIC in diabetics with severe microangiopathy regardless of the duration of the disease and type of diabetes. When CIC were detected by RIA, they were found to be at a higher prevalence in diabetics than in normal controls but no correlation was noted with the occurrence of microangiopathy. CIC, detected by RIA, were higher in long standing Type1 diabetics. These differences in the results can be explained by the view that CIC present in diabetics seems are heterogeneous in nature with a proportion of them being related to the occurrence of microangiopathy (Adreani *et al.*, 1982). Thus, as reported herein, diabetics with microangiopathy showed increased levels of CIC as compared to controls. These results are similar to the results obtained by RIA. In addition, we also noted a correlation between microalbuminuria (a microvascular complication) and levels of CIC IgG.

CIC differ in size which is determined by the ratio of antigen–antibody, the valence and nature of antigen and the affinity and class of antibodies. Based on size the large CIC appear as insoluble proteins which are rapidly cleared from the circulation facilitated by mononuclear phagocytes and therefore, do not accumulate along vessel walls. Presence of a large excess of the auto-antigen leads to the formation of small CIC. The smaller CIC are thus more harmful since they are deposited in the small blood vessels of the microcirculation. Such deposition of CIC results in microangiopathy, and in our case microalbuminuria. There is evidence that proteins, including immunoglobulins, accumulate in the matrix lining of the walls of small blood vessels in diabetic subjects (Wilson *et al.*, 1991). This is supported by the fact that the amount of IgG cross-linked to the glomerular basement membrane is five times higher in diabetic than in non diabetic rats (Brownlee *et al.*, 1983). IgG binds preferentially to the basement membranes, which leads to accelerated non enzymatic glycation of proteins and advanced glycation end products (AGE, Brownlee *et al.*, 1983).

A previous study reported levels of CIC in 57 diabetic patients (Baranao *et al.*, 1985). In these studies the levels of CIC were quantitated by the binding of CIC to human red blood cells through the C3b complement fraction, a radioimmunoassay using $^{125}$I labeled anti-human IgG (HRBC RIA test) and CIC precipitation using 3.5% PEG (PEG test). Patients were divided in two groups according to the type of diabetes. Their results showed that CIC levels were significantly higher in both groups of patients when compared to normal controls. Type 1 diabetes patients with microangiopathy present higher CIC levels as measured by HRBC RIA test than Type 1 patients without microvascular complications, while no difference was found within the Type 2 group.

There have also been reports for antibodies against C1q complement component in the sera of patients with different diseases. These antibodies are one of the reasons for false positive results as detected by using the C1q-ELISA (Haseley *et al.*, 1997; Walport *et al.*, 1998). Therefore it is possible that a part of the C1q-ELISA positive results was due to such antibodies and not to CIC. In contrast to these assays, the assay described herein utilized a protein isolated from a parasitic plant which minimizes the potential of cross-reactivity (Stanilova and Slavov, 2001). In addition, ELISA is a highly sensitive and specific immunological assay. It is easier to accomplish and cheaper than radioimmunological tests which not only require specialized equipment for work involving radioactive substances but is considered ecologically unsafe.

An additional two fundamental risk factors associated with microalbuminuria need address. This includes elevated blood pressure and elevated serum glucose concentration. The significance of lipid profile (elevated concentration of triglycerides and total cholesterol) also needs to be taken into account (Microalbuminuria Collaborative Study Group, United Kingdom, 1993; Poulsen *et al.*, 1994; Mortensen and Poulgaard, 1997). An increase in the levels of plasma triglycerides which are a rich lipoprotein species that are potentially atherogenic has expanded the picture of diabetic dyslipidaemia (Taskinen, 2003). It is the most common abnormality found in poorly controlled diabetes. In CIC positive patients with microvascular complications, the binding of the triglycerides to the arterial wall proteins may convert these proteins into an immunogenic form. In the present study, tryglycerides were over the normal limit in the group of patients with vascular complications supporting this view.

The accelerated non-enzymatic glycation of proteins may also potentially be a reason for the formation of new epitopes. Advanced glycation of protein may thus increase immunogenicity. The fact that AGEs has shown to possess antigenic properties has led to the hypothesis that the physico-biochemical structure of AGE may change sufficiently to be unique enough to induce an autoimmune response (Turk *et al.*, 2001). It is possible that some of CIC detected in the studies reported herein contain AGE and anti AGE complexes. Turk *et al.* (2001) reported that the content of AGE in soluble immune complexes was significantly higher in the sera of diabetic patients than in control subjects and correlated inversely with the level of AGE specific free antibodies. Interactions of AGE autoantibodies with AGE as a continuously produced antigen result in the formation of AGE-immune complexes that may play a role in the atherogenic process (Turk *et al.*, 2001). The development of microvascular disease is associated with high percentage of glycated hemoglobin (HbA1c). In this study CIC IgG of diabetic group 1 correlated with HbA1c. HbA1c is a classical example of AGE. These results support the hypothesis for accelerated non-enzymatic glycation.

Our results showed that the highest levels of CIC were of the IgG isotype followed by IgM and then IgA. The formation of IgM antibodies precedes the development of IgG antibodies against a given immunogen by a process termed Ig class switching. The elevation of CIC of the IgM and IgG types is therefore the first indicator of pathological turnover and the development of microvascular complications in diabetic children. The highest percent of patients with elevated levels of CIC IgM was found in group 2 (61%). It is of interest to study whether these children will go on to develop microvascular complications, and as such CIC IgM may be used as a predictor of diabetic vascular damage. We did not find significant elevation of CIC IgA in patients. The low frequency of patients with CIC IgA (9%) may be explained by the fact that IgA appears during the later stages of the disease. It is important to note that the highest levels of CIC IgG was seen in one patient who had elevated blood pressure and the other had microalbuminuria. Triolo *et al.* (1984) examined the sera of middle age Type 1 and Type 2 diabetics for the presence of IgA- and insulin-containing CIC using a solid-phase anti-C3 enzyme immunoassay. CIC IgM and CIC IgG were also investigated. The patients groups were divided according to the treatment. The authors found CIC IgA to be associated with the presence of microangiopathy, suggesting that they may play a role in the pathogenesis of the late diabetic complications (Triolo *et al.*, 1984). In the present study we also investigated CIC IgM, CIC IgG and CIC IgA, but the results showed an association of CIC IgG with microangiopathy. This contradiction between our results could be due to the different ages of the two study populations, the type of diabetes, the methodology utilized for the assays utilized.

Most of our patients with vascular complications had microalbuminuria. Only three patients were reported to have high blood pressure without microalbuminuria. The retinopathy or high blood pressure was developed after the onset of microalbuminuria.

The prevalence of microalbuminuria in Type1 diabetic patients is a sensitive predictor of future development of diabetic nephropathy. Diabetic nephropathy is the leading cause of end-stage renal disease. Because early diagnosis and treatment may prevent the complication, new tools for early detection are also needed. Microalbuminuria is a recognized risk factor for increased mortality and renal failure in Type 1 diabetes (Kate and Walker, 2003). Risk factors for the development of nephropathy include positive family history, male sex, poor glycemic control, hypertension, smoking and the presence of retinopathy and coronary artery disease. The pathogenesis of glomerulonephritis involves three phases of injury. The first is usually an antigen-antibody reaction that secondarily activates one or more chemical mediators and stimulates resident cells to proliferate and/or transform metabolically. At this point the glomerulus is not irreversibly damaged and can heal. The third phase is progression to chronic disease due to buildup of glomerular basement membrane material resulting in sclerosis and an obliteration of the surface area responsible for ultrafiltration.

The passage of proteins across the glomerular filtration barrier is regulated by several factors. Small proteins, such as albumin, filter through approximately 5.5 nm of glomerular pores, while immunoglobulins, because of their size (5.5 nm), can only escape across the larger shunt pores of alternative filtration pathways (size-selectivity) (Brenner *et al.*, 1978). The structural basis for the development of nephropathy is the slow expansion of the volume of the glomerular mesangial matrix which results in increased glomerular permeability to proteins, decreased filtration surface and hypertension. Eventually the glomeruli becomes completely scarred. The development of nephropathy and retinopathy are closely linked. However, microalbuminuria was not a good marker for diabetic retinopathy (Romero *et al.*, 2003).

The first manifestation of diabetic nephropathy is microalbuminuria, which evolves into asymptomatic overt proteinuria and later the nephrotic syndrome. Hypertension often accompanies the development of proteinuria and worsens as renal function declines. No gene for diabetic nephropathy has so far been identified. Microalbuminuria not only implies the presence of renal involvement in diabetes mellitus, but also is associated with generalized vascular damage.

In conclusion, we suggest that elevated levels of CIC IgG are associated with the development of early diabetic nephropathy in children with Type 1 diabetes. Although it has been shown that metabolic control influences the development of microvascular disease (Klein *et al.*, 1988), it now appears very probable that an immunological component is also involved in the pathogenesis of microangiopathy. Elucidation of the mechanism whereby immunoglobulins influence the development of microvascular complication will require intensive research. Identification of a subset of patients who are immunologically more susceptible to developing microvascular complication may provide clinicians a tool that can be utilized to control the development of such microvascular complications and thus disease course. Although the precise mechanisms by which soluble IC induce pathological damage in diabetics have not been fully elucidated, we have obtained sufficient evidence to prove that antigen-antibody complexes exist in diabetics which are associated with higher frequencies of complications.

### *References*

Adreani, D., Di Mario, U., Galfo, C., *et al.* (1982) "Circulating immune complexes in diabetics with severe microangiopathy: evaluation by two different methods", *Acta Endocrinol.* **99**, 239–244.

Allen, K.V. and Walker, J.D. (2003) "Microalbuminuria and Mortality in Long-Duration Type 1 Diabetes", *Diabetes Care* **26**, 2389–2391.

Baranao, R.I., Tesone, P.A., Carberi, J.C., *et al.* (1985) "Circulating immune complexes and B-lymphocytes in diabetic patients with and without microangiopathy", *Acta Physiol. Pharmacol. Latinoam.* **35**, 145–151.

Baydanoff, S., Nicoloff, G., Russev, T., *et al.* (1988) "Detection of elastin-antielastin circulating immune complexes (CIC) in diabetic patients with vascular damage", *Cor. Vasa.* **30**, 361–367.

Bottazzo, G.F. (1993) "Banting Lecture. On the honey disease. A dialogue with Socrates", *Diabetes* **42**, 778–800.

Brenner, B.M., Hostetter, T.H. and Humes, H.D. (1978) "Molecular basis of proteinuria of glomerular origin", *N. Engl. J. Med.* **298**, 826–833.

Brownlee, M., Pongar, S. and Cerami, A. (1983) "Covalent attachment of soluble proteins by nonenzymatically glycosylated collagen", *J. Exp. Med.* **158**, 1739–1744.

Di Mario, U., Ventriglia, L., Yavicoli, M., *et al.* (1983) "The correlation between insulin antibodies and circulating immune complexes in diabetics with and without microangiopathy", *Clin. Exp. Immunol.* **52**, 575–580.

Haseley, L.A., Wisnieski, J.J., Denburg, M.R., *et al.* (1997) "Antibodies to C1q in systemic lupus erythematosus: characteristics and relation to Fc gamma RIIA alleles", *Kidney Int.* **52**, 1375–1380.

Klein, R., Klein, B.E.K., Moss, S.E., *et al.* (1988) "Glycosylated hemoglobin predicts the incidence and progression of diabetic retinopathy", *JAMA* **260**, 2864–2871.

Linderkamp, O., Ruef, P., Zilow, E., *et al.* (1999) "Impaired deformability of erythrocytes and neutrophilis in children with newly diagnosed insulin-dependent diabetes mellitus", *Diabetologia* **43**, 865–869.

Microalbuminuria Collaborative Study Group, United Kingdom (1993) "Risk factors for development of microalbuminuria in insulin dependent diabetic patients: a cohort study: Microalbuminuria Collaborative Study Group, U.K.", *BMJ* **306**, 1235–1239.

Mortensen, H.B. and Poulgaard, P. (1997) "Hvidore Study Group on Childhood Diabetes. Comparison of metabolic control in a cross-sectional study of 2.873 children and adolescents with IDDM from 18 countries", *Diabetes Care* **20**, 714–720.

Nicoloff, G., Baydanoff, S., Stanimirova, N., *et al.* (2000) "Relationship between elastin-derived peptides and the development of diabetic microvascular complications—a longitudinal study in children with Type 1 (insulin dependent) diabetes mellitus", *General Pharmacol.* **35**, 59–64.

Nicoloff, G., Baydanoff, S., Stanimirova, N., *et al.* (2000) "An association of anti-elastin IgA antibodies with development of retinopathy in diabetic children", *General Pharmacol.* **35**, 83–87.

Nicoloff, G., Baydanoff, S., Stanimirova, N., *et al.* (2001) "Detection of collagen type IV in children with Type 1 (insulin dependent) diabetes mellitus—a longitudinal study", *Pediatr. Diabetes* **2**, 184–190.

Nicoloff, G., Baydanoff, S., Petrova, C., *et al.* (2002) "Serum antibodies to collagen type IV and development of diabetic vascular complications in children with type 1 (insulin-dependent) diabetes mellitus. A longitudinal study", *Vasc. Pharmacol.* **38**, 143–147.

Ölmez, U., Garred, P., Mollnes, T.E., *et al.* (1991) "C3 activation products, C3 containing immune complexes, the terminal complement complexes and native C9 in patients with rheumatoid arthritis", *Scand. J. Rheumatol.* **20**, 183–189.

Poulsen, P.L., Hansen, K.W. and Mogensen, C.E. (1994) "Ambulatory blood pressure in the transition from normo-to microalbuminuria. A longitudinal study in IDDM patients", *Diabetes* **43**, 1248–1253.

Romero, P., Salvat, M., Mendez, M., *et al.* (2003) "Is microalbuminuria a risk factor for diabetic retinopathy?", *J. Fr. Ophtalmol.* **26**, 680–684.

Stanilova, S.A. and Slavov, E.S. (2001) "Comparative study of circulating immune complexes quantity detection by three assays—CIF-ELISA, C1q-ELISA and anti-C3 ELISA", *J. Immunol. Methods* **253**, 13–21.

Stanilova, S. and Slavov, E. (2003) "New ELISA kits using C3 binding glycoprotein from Cuscuta europea detect mainly IgM CIC in rheumatoid arthritis and progressive systemic lupus erythematosus", *Clin. Develop. Immunol.* **10**, 111–119.

Taskinen, M.R. (2003) "Diabetic dyslipidaemia: from basic research to clinical practice", *Diabetologia* **46**, 733–749.

Triolo, G., Giardina, E., Rinaldi, A., *et al.* (1984) "IgA- and insulin-containing (C3-fixing) circulating immune complexes in diabetes mellitus", *Clin. Immunol. Immunopathol.* **30**, 169–177.

Turk, Z., Ljubic, S., Turk, N., *et al.* (2001) "Detection of autoantibodies against advanced glycation endproduct and AGE-immune complexes in serum of patients with diabetes mellitus", *Clinica Chimica Acta* **303**, 105–115.

Verge, C.F., Stenger, D., Bonifacio, E., *et al.* (1998) "Combined use of autoantibodies IA-2 autoantibody, GAD autoantibodies, insulin autoantibodies, cytoplasmic islet cell autoantibodies in type 1 diabetes: combinatorial islet autoantibody workshop", *Diabetes* **47**, 1857–1866.

Walport, M.J., Davies, K.A. and Botto, M. (1998) "C1q and systemic lupus erythematosus", *Immunobiology* **199**, 265–285.

Wilson, C., Fornasieri, A., Moullier, P., *et al.* (1991) "Renal diseases", In: Stites, D. and Terr, A., eds, Basic and clinical immunology, 7th Edn. (Appletion & Lange Norwalk, CT/San Mateo, CA), pp 526–538.

Zhelev, Z.H., Stanilova, S. and Carpenter, B. (1994) "Isolation, partial characterization and complement inhibiting activity of a new glycoprotein from Cuscuta europea", *Biochem. Biophys. Res. Commun.* **202**, 186–196.

